# Saúde mental infantojuvenil: análise de itinerários terapêuticos em município de interior e sem Centros de Atenção Psicossocial Infantojuvenis

**DOI:** 10.1590/0102-311XPT115824

**Published:** 2025-02-07

**Authors:** Iagor Brum Leitão, Luziane Zacché Avellar, Thays Picoli Martins, Júlia Miloti, Tharssa Karolynie da Silva Negreiros Fernandes

**Affiliations:** 1 Universidade Federal do Espírito Santo, Vitória, Brasil.; 2 Centro Universitário Vale do Cricaré, São Mateus, Brasil.

**Keywords:** Itinerário Terapêutico, Saúde Mental, Criança, Adolescente, Therapeutic Itinerary, Mental Health, Child, Adolescent, Ruta Terapéutica, Salud Mental, Niño, Adolescente

## Abstract

Pesquisas sobre itinerários terapêuticos em saúde mental infantojuvenil têm sido conduzidas predominantemente em Centros de Atenção Psicossocial Infantojuvenis (CAPSij) ou em regiões equipadas com esses serviços. Visando expandir a compreensão desse campo, este estudo analisou os itinerários terapêuticos de crianças e adolescentes atendidos pelo serviço de Equipe Multiprofissional em Saúde Mental - localmente conhecido como “Saúde Mental” - em um município de interior e sem CAPSij. Realizamos entrevistas semiestruturadas com 12 mães e dois pais, visando compreender as motivações para as buscas por cuidados, os locais percorridos e as experiências durante esse processo. Os dados foram analisados por meio da classificação hierárquica descendente, possibilitada pelo IRaMuTeQ. Quatro categorias foram formadas: (1) detecção inicial de necessidades; (2) motivações para busca de atendimento; (3) dinâmica do serviço; e (4) fatores que afetam a continuidade do cuidado. As escolas prevaleceram como as impulsionadoras da busca por cuidados, enquanto as unidades básicas de saúde atuaram mais como pontos de encaminhamento para especialistas. Psicológos(as) e psiquiatrias da Equipe Multiprofissional em Saúde Mental, fonoaudiólogos da Policlínica, e neurologistas da rede privada e do Centro Regional de Especialidades foram as especialidades mais frequentadas. A alta rotatividade de profissionais foi tida como a principal barreira ao acesso e à continuidade do cuidado. Ao contrário de outros estudos desenvolvidos em contextos com redes de serviços mais abrangentes, que frequentemente sugerem fortalecer as redes existentes de saúde mental infantojuvenil, este estudo destaca a necessidade de construir tais redes desde o início.

## Abstract (en)

Research on therapeutic itineraries in child and adolescent mental health has been predominantly carried out in Child and Adolescent Psychosocial Care Centers or in regions that have these services. To expand the understanding of this field, this study analyzed the therapeutic itineraries of children and adolescents assisted by the Multiprofessional Mental Health Team service - locally known as “Mental Health” - in an inner municipality without Child and Adolescent Psychosocial Care Centers. Semistructured interviews were conducted with 12 mothers and two fathers to understand their motivations for seeking care, the places they visited, and the experiences they had in this process. The data were analyzed by descending hierarchical classification, made possible by IRaMuTeQ. In total, four categories were formed: (1) initial detection of needs, (2) motivations for seeking care, (3) service dynamics, and (4) factors that affect continuity of care. Schools prevailed as the drivers of the search for care, whereas basic health units acted more as referral points for specialists. Psychologists and psychiatrists from the Multiprofessional Mental Health Team, speech therapists from the Polyclinic, and neurologists from the private network and the Regional Center of Specialties were the specialties that received the most visits. Participants deemed the high turnover of professionals as the main barrier to access and continuity of care. Unlike other studies - which have been developed in contexts with more comprehensive service networks -, which often suggest strengthening the existing child and adolescent mental health networks, this study highlights the need to build such networks from the ground up.

## Abstract (es)

La investigación sobre itinerarios terapéuticos en salud mental infantojuvenil se realiza predominantemente en Centros de Atención Psicosocial Infantojuvenil (CAPSij) o en regiones que proporcionan estos servicios. Con el objetivo de ampliar la comprensión de este campo, este estudio analizó los itinerarios terapéuticos de niños y adolescentes atendidos por el servicio Equipo Multiprofesional en Salud Mental -conocido localmente como “Salud Mental”- en un municipio del campo y sin CAPSij. Se realizaron entrevistas semiestructuradas a 12 madres y dos padres, con el fin de identificar las razones para la búsqueda de atención, los lugares recorridos y las experiencias durante este proceso. Los datos se analizaron utilizando la clasificación jerárquica descendente por IRaMuTeQ. Se formaron cuatro categorías: (1) detección inicial de necesidades, (2) razones para buscar atención, (3) dinámica de servicios, y (4) factores que afectan la continuidad de la atención. Las escuelas prevalecieron como promotoras de la búsqueda de atención, mientras que las unidades básicas de salud actuaron más como puntos de derivación para las especialidades. Los especialistas más buscados fueron psicólogos(as) y psiquiatras del Equipo Multiprofesional en Salud Mental, fonoaudiólogos de la Policlínica y neurólogos de la red privada y del Centro Regional de Especialidades. La alta rotación de profesionales se consideró la principal barrera para el acceso y la continuidad de la atención. A diferencia de otros estudios desarrollados en contextos con redes de servicios más integrales, que frecuentemente llaman la atención para el fortalecimiento de las redes existentes de salud mental infantojuvenil, este estudio destaca la necesidad de construir dichas redes desde el principio.

## Introdução

A implementação de uma política de saúde mental infantojuvenil no Brasil é recente. Historicamente, as ações direcionadas a crianças e adolescentes em sofrimento psíquico eram baseadas em práticas institucionalizantes, frequentemente filantrópicas, e que visavam responder aos problemas de pobreza e abandono ^1^. O marco legal para a garantia de direitos tem seu início na década de 1990, com a promulgação do *Estatuto da Criança e do Adolescente* ^2^, enquanto o reconhecimento da saúde mental infantojuvenil no âmbito das políticas públicas é uma conquista iniciada somente nas últimas duas décadas ^3^ ^,^ ^4^ ^,^ ^5^.

A perspectiva de crianças e adolescentes em sofrimento psíquico como sujeitos de direitos e desejos, sendo, portanto, implicados em sua demanda por cuidado em saúde mental, propõe uma ruptura com as práticas que compunham o paradigma dominante na saúde. Esse paradigma tende a ser orientado pela tutela, segregação e, sobretudo, por uma abordagem medicalizante do sofrimento psíquico ^5^.

Apesar dos avanços regulatórios e políticos, a situação da rede para o cuidado em saúde mental infantojuvenil tem recebido críticas quanto à insuficiência de serviços e falta de profissionais capacitados e suportados para esse tipo de trabalho, de acordo com os preceitos da política, que envolve um cuidado ampliado, corresponsável e intersetorial ^6^. No entanto, estudos na área têm sido crescentes, abrangendo uma variedade de desenhos metodológicos e objetos de análise. Eles analisam o perfil epidemiológico e sociodemográfico dos usuários atendidos, as ações de cuidado, os processos de trabalho, as potencialidades e os desafios vividos, assim como as barreiras enfrentadas por usuários e familiares durante as buscas pelo cuidado ^7^ ^,^ ^8^.

Nesse contexto, destacam-se os estudos sobre itinerários terapêuticos. Conceitualmente, referem-se a todos os caminhos percorridos por indivíduos e grupos na tentativa de solucionar seus problemas de saúde ^9^. Mesmo em municípios com um quantitativo maior de serviços de saúde mental infantojuvenil, são identificados problemas no acesso e na intersetorialidade do cuidado ^6^. Pesquisas indicam que esse cenário tende a se acentuar em municípios menores ou do interior ^10^ ^,^ ^11^.

Dado o exposto, este estudo analisou os itinerários percorridos por famílias de crianças e adolescentes atendidas pelo serviço de Equipe Multiprofissional em Saúde Mental - localmente conhecido como Saúde Mental - de um município do interior brasileiro sem Centros de Atenção Psicossocial Infantojuvenis (CAPSij). Buscou-se entender as motivações para as buscas por cuidados em saúde mental, os locais percorridos e as experiências vividas durante esse processo.

A relevância deste estudo está na aplicação de uma metodologia que permite informar sobre as interações dos familiares com os sistemas de saúde mental infantojuvenil, contribuindo com o desenvolvimento de políticas públicas e orientando as ações de gestores. Identificar os caminhos percorridos para o acesso ao cuidado é essencial para favorecer esse acesso, qualificar e sustentar as ações de cuidado em rede, fortalecer essa rede e promover o cuidado territorial. Essa relevância é ainda mais destacada pelo fato de que a maioria das pesquisas sobre itinerários terapêuticos de saúde mental infantojuvenil tem sido conduzida com usuários referenciados por CAPSij ^12^, evidenciando uma ausência de estudos sobre itinerários terapêuticos em regiões que não dispõem de CAPSij.

## Método e cenário da pesquisa

Estudo descritivo de abordagem qualitativa conduzido por meio de entrevistas semiestruturadas. Foi desenvolvido com familiares de crianças e adolescentes que estavam recebendo atendimento no serviço Saúde Mental, localizado no prédio de uma Policlínica de um município do interior de um estado do Sudeste do Brasil. O município em questão tem uma população estimada em 130 mil habitantes. Na região, o serviço é popularmente conhecido como “Saúde Mental”.

As Equipes Multiprofissionais em Saúde Mental são serviços especializados registrados no Cadastro Nacional de Estabelecimentos de Saúde (CNES). São situados no âmbito secundário da saúde, sendo responsáveis pela oferta de cuidado individual e grupal, sempre em articulação com outros pontos da Rede de Atenção Psicossocial (RAPS), em complementaridade coordenada com os demais serviços de saúde mental no território ^13^.

No município, o serviço Saúde Mental está operando desde o ano de 2015 e ainda está em processo de habilitação formal pelo Ministério da Saúde. No entanto, compõe a RAPS do município junto com um Centro de Atenção Psicossocial (CAPS I), um Centro de Atenção Psicossocial Álcool e Drogas (CAPS AD), 28 Unidades Básicas de Saúde (UBS), um Centro Regional de Especialidades (CRE), um Centro de Referência das Juventudes (CRJ), entre outros serviços de saúde, como um Hospital Geral estadual e um municipal, uma Unidade de Pronto Atendimento (UPA) e uma Policlínica. Conforme informado pela coordenação do serviço Saúde Mental, houve uma pactuação entre os gestores da RAPS em designar a unidade para a realização de atendimentos especializados em saúde mental infantojuvenil. Quando ocorreu a coleta de dados, entre novembro de 2023 e março de 2024, o serviço Saúde Mental alocava atendimentos dedicados às crianças e aos adolescentes uma vez por semana. Nos outros dias, a equipe atendia preferencialmente adultos com demandas de saúde mental não associadas a condições graves, mantendo uma separação entre os diferentes grupos de pacientes. Por configurar o único serviço autodeclarado para o cuidado em saúde mental infantojuvenil, a Equipe Multiprofissional em Saúde Mental foi escolhida como cenário para esta pesquisa de itinerários terapêuticos.

### Participantes

O critério de inclusão foi: familiar ou cuidador responsável pela criança ou adolescente que estivesse em atendimento pelo serviço Saúde Mental há, no mínimo, um mês. No entanto, ao longo da entrevista, os dados poderiam ser excluídos caso fosse observado que o familiar não possuía conhecimento suficiente do caso para fornecer detalhes sobre os itinerários terapêuticos da criança ou do adolescente, bem como informações relevantes sobre seu histórico. Os participantes foram abordados e convidados a participar do estudo durante suas visitas ao serviço. No total, 14 genitores participaram do estudo, sem que houvesse necessidade de exclusão de entrevista de nenhum participante.

### Instrumentos, procedimentos de coleta e análise de dados

O roteiro da entrevista foi semiestruturado. Explorou questões sobre as motivações para a busca de cuidados em saúde mental infantojuvenil, as trajetórias percorridas por cada família até o acesso ao serviço Saúde Mental e os possíveis destinos subsequentes, bem como a experiência vivida durante a busca pelo cuidado. Também contemplou itens sociodemográficos das crianças ou adolescentes em atendimento, como sexo, idade, cor de pele, possíveis diagnósticos ou condições médico-psiquiátricas.

As entrevistas foram conduzidas em salas reservadas do próprio serviço, tiveram duração média de 50 minutos, foram gravadas em áudio e transcritas. A coleta foi encerrada quando se atingiu a saturação dos dados, indicando que novas entrevistas não forneceriam informações adicionais significativas.

As transcrições das entrevistas foram compiladas em um único *corpus* textual. Análises léxicas foram realizadas por meio da plataforma Interface de R para Análises Multidimensionais de Textos e Questionários (IRaMuTeQ; http://www.iramuteq.org/). A primeira técnica de análise utilizada foi a classificação hierárquica descendente (CHD), que examina as raízes lexicais e o contexto de formação das classes dentro do *corpus*. A CHD emprega o teste qui-quadrado (χ^2^) para identificar a força associativa entre as palavras em suas respectivas classes. Após o processamento dos dados, o programa gera um dendrograma que ilustra as classes obtidas ^14^.

A segunda análise empregada foi a análise de similitude (AS). Diferentemente da CHD, a AS permite a seleção manual de termos específicos do *corpus*, possibilitando que o programa trace e exiba graficamente as conexões entre eles. Essa técnica foi utilizada para representar os caminhos tomados por familiares e usuários. Foram selecionados somente termos que indicassem as ações de busca (como “ir”, “buscar”, “procurar”, “encaminhar”), associados aos locais ou aos profissionais frequentados (como “psicólogo”, “saúde_mental”, “aqui”, “policlínica”, “escola”, “posto”) e relacionados ao que foi procurado nesses locais ou ao que foi conduzido (como “tratamento”, “consulta”, “exame”, “relatório”, “receita”, “laudo”).

### Procedimentos éticos

A pesquisa foi aprovada pelo Comitê de Ética em Pesquisa da Universidade Federal do Espírito (CAAE 67659022.6.0000.5542).

## Resultados

Participaram das entrevistas 14 genitores de crianças e adolescentes atendidos no serviço Saúde Mental, sendo 12 mães e dois pais. Duas mães relataram ter dois filhos cada em atendimento no serviço, totalizando 16 crianças e adolescentes. Conforme descrito no Quadro 1, observa-se que as idades dos usuários variaram de 3 a 16 anos, com média de 9,56 anos. A maioria era negra (n = 15), do sexo masculino (n = 13), crianças (n = 11) e matriculados no Ensino Fundamental I (n = 11).

**Quadro 1:** Características sociodemográficas dos participantes.

PARTICIPANTE	FILHOS EM ATENDIMENTO EM SAÚDE MENTAL	SEXO DO USUÁRIO	IDADE DO USUÁRIO (ANOS)	COR DE PELE DO USUÁRIO	ESCOLARIDADE DO USUÁRIO	CONDIÇÃO MÉDICA/PSIQUIÁTRICA DO USUÁRIO E/OU MOTIVO DE BUSCA PARA ATENDIMENTO EM SAÚDE MENTAL
Mãe 1	2	Ambos do sexo masculino	10 e 7	Ambos pretos	5º ano e 1º ano (Ensino Fundamental I)	Criança de 10 anos: investigando déficit de atenção e motivo de “crises de ansiedade” Criança de 7 anos: investigando motivos de inquietação e dificuldades escolares
Mãe 2	1	Masculino	8	Preto	3º ano (Ensino Fundamental I)	Diagnóstico de TDAH e investigação de TEA
Mãe 3	1	Masculino	3	Preto	Pré-escola	Diagnóstico de TEA
Mãe 4	2	Ambos do sexo masculino	Ambos com 7 anos (gêmeos)	Ambos pardos	Ambos no 2º ano (Ensino Fundamental I)	Ambos em investigação de TDAH
Mãe 5	1	Masculino	6	Preto	Pré-escola	Diagnóstico de TEA e investigação de TDAH e epilepsia
Mãe 6	1	Feminino	8	Pardo	3º ano (Ensino Fundamental I)	“Sentimento de inferioridade”
Mãe 7	1	Masculino	12	Preto	7º ano (Ensino Fundamental II)	Investigação de TDAH e TEA
Mãe 8	1	Masculino	10	Preto	5º ano (Ensino Fundamental I)	Diagnóstico de TDAH
Mãe 9	1	Masculino	16	Branco	1º ano (Ensino Médio)	Diagnóstico de TEA e TDAH
Mãe 10	1	Masculino	12	Pardo	6º ano (Ensino Fundamental II)	Diagnóstico de TDAH e investigação de TEA
Mãe 11	1	Masculino	15	Preto	1º ano (Ensino Médio)	Diagnóstico de TEA e depressão
Mãe 12	1	Feminino	7	Parda	2º ano (Ensino Fundamental I)	Diagnóstico de TDA
Pai 1	1	Masculino	11	Pardo	5º ano (Ensino Fundamental I)	Diagnóstico de TDAH e TEA
Pai 2	1	Feminino	14	Parda	9º ano (Ensino Fundamental II)	Diagnóstico de “depressão psicótica”

TDA: transtorno de déficit de atenção; TDAH: transtorno de déficit de atenção com hiperatividade; TEA: transtorno do espectro autista.

Fonte: dados da pesquisa.

Entre os diagnósticos psiquiátricos relatados pelos genitores, destacaram-se o transtorno do déficit de atenção com hiperatividade (TDAH) e transtorno do espectro autista (TEA). Segundo os relatos, sete usuários estavam em processo de investigação diagnóstica, abrangendo principalmente TDAH, TEA e epilepsia. Outros casos envolviam queixas de baixa autoestima e dificuldades escolares e comportamentais.

**Figura 1:**
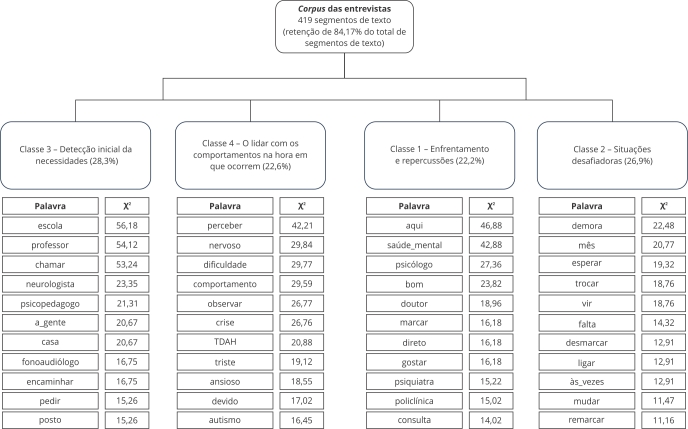
Dendrograma editado com base no resultado da análise da classificação hierárquica descendente (CHD), gerado pelo IRaMuTeQ, utilizando o *corpus* textual composto pela transcrição das entrevistas com os 14 genitores participantes.

A CHD formou quatro classes, conforme apresentado no dendrograma exibido na Figura 1. O *corpus*, composto por transcrições de 14 entrevistas, incluiu aproximadamente 17 mil palavras e 27 páginas de transcrição. No processo de análise, o *corpus* foi dividido em 498 segmentos de texto, dos quais 419 (84,17%) foram retidos para análise pela CHD. Uma análise eficaz utilizando a CHD requer a retenção de, no mínimo, 75% dos segmentos de texto ^14^.

Na Figura 1, a partição à direita resultou nas classes 1 e 2 e, na partição à esquerda, originaram-se as classes 3 e 4.

### Detecção inicial das necessidades

Começando pela partição à esquerda, a classe 3 foi intitulada de “Detecção inicial das necessidades” e compôs 28,3% dos segmentos de texto analisados. Agregou termos que indicam as primeiras observações e ações tomadas pelos agentes mais próximos às crianças e aos adolescentes, geralmente pela escola e família, em que os primeiros sinais notados motivaram a busca por atendimentos.

Os termos “escola” e “professor” indicaram que o ambiente educacional desempenhou papel crucial para a busca por atendimento. “Chamar” representou a ação tomada após a observação de sinais tido como preocupantes: chamar a família, designada pela expressão “a_gente”, e sugerir a busca por atendimentos especializados. “Neurologista”, “psicopedagoga” e “fonoaudiólogo” representaram alguns dos profissionais sugeridos. No sistema público do município, o profissional neurologista atua no CRE; contudo, devido às longas filas de espera, a maioria dos participantes relatou ter recorrido ao sistema particular, inclusive para a realização de exames. O fonoaudiólogo atua na Policlínica do município, e o psicopedagogo foi encontrado tanto nas escolas de origem quanto em serviços privados.

Por sua vez, o termo “casa” indicou que determinados sinais também foram observados pela própria família. Já o termo “posto” se referiu à participação das UBS no processo, como locais buscados pela família para “pedir” encaminhamentos para especialistas.

“*Por conta da escola, eu fui procurar o especialista, porque a professora, nas reuniões, falou assim: ‘talvez ele precise de um fonoaudiólogo, que vai passar algumas atividades de concentração, essas coisas assim’*” (Mãe 1).

“*A própria escola pediu para estar investigando, ele já passou pelo neurologista e agora na verdade ele precisa passar pela fonoaudióloga, pois tem problema na dicção também, na fala*” (Pai 1).

“*Foi de lá do postinho que eu vim, que eu fui primeiro na pediatra já nesse sentido de falar que ela precisava desse encaminhamento, e depois eu já vim para aqui* [serviço Saúde Mental]” (Mãe 6).

### Motivações para busca de atendimento

A classe 4, denominada “Motivações para busca por atendimento”, representou 22,6% dos segmentos de texto. Destacaram-se os termos “nervoso”, “comportamento”, “dificuldade”, “crise”, “autismo”, “ansioso” e “TDAH”, os quais apareceram associados aos sinais observados que mais conduziram os familiares a procurarem atendimento em saúde mental infantojuvenil.

“*É porque ele tem algumas dificuldades mentais. Na escola mesmo, ele tem dificuldade de aprendizagem, não consegue desenvolver as coisas e tudo tem que passar pela professora*” (Pai 1).

“*E aí, na escola, começaram a me questionar sobre o comportamento dele, com alguns comportamentos fora do comum, e aí passei a procurar*” (Mãe 5).

“*Eu não pensava que era autismo, aí quando eu fui no psiquiatra, muito atencioso com a_gente, ele pegou e falou comigo que ele iria ser uma criança especial e que tudo indicava que era um autismo*” (Mãe 11).

### Dinâmica do serviço Saúde Mental

Já no que se refere à partição da direita, a classe 1, intitulada “Dinâmica do serviço Saúde Mental”, representou 26,9% dos segmentos de texto. Os termos “marcar” e “direto” indicaram como muitos participantes chegaram ao serviço: por meio de agendamentos, com alguns casos sem passar por outros locais. O termo “consulta” esteve associado aos termos “psicólogo”, “doutor” e “psiquiatra”. Já a presença do termo “bom” e “gostar” se referiu à avaliação positiva dos profissionais.

“*Comecei a trabalhar na loja e tive plano_de_saúde. Daí eu fui tentar procurar pelo plano_de_saúde, mas também não saiu. Consegui quatro sessões de psicólogo, mas não saía a consulta. Só estava a sessão carimbada, mas tinha que esperar sair para marcar a consulta. Então, para mim, a primeira consulta com psicólogo dele foi aqui* [no serviço Saúde Mental]” (Mãe 1).

“*A psicóloga* [do serviço Saúde Mental] *que atendia a_gente era ótima, fazia um atendimento bom, até que ela saiu*” (Mãe 3).

“*No primeiro momento, consegui marcar* [no serviço Saúde Mental]*. Marquei, mas marcaram na quinta-feira, sendo que na quinta-feira, é adulto que atende. O médico só atende adulto, criança não*” (Mãe 5).

### Fatores que afetam a continuidade do cuidado

A classe 2, intitulada “Fatores que afetam a continuidade do cuidado”, compôs 25,9% dos segmentos de texto. Agrupou uma variedade de termos que refletiram os desafios enfrentados pelos usuários dos serviços de saúde.

Os termos “demorar”, “esperar” e “mês” estiveram relacionados às longas esperas para obter atendimento especializado, especialmente com o profissional médico neurologista pediatra. Os termos “vir”, “às_vezes”, “desmarcar”, “ligar” e “remarcar” refletiram aspectos negativos de alguns serviços, criticados por falhas na comunicação, especialmente com relação a cancelamentos não notificados de consultas, que resultavam em visitas perdidas. Já os termos “trocar” e “mudar” apontam para a alta rotatividade de profissionais, sentidas como prejudiciais para a continuidade e a confiança no cuidado.

“*Porque muda de governo e acaba mudando também os médicos, e a_gente não tem noção*” (Mãe 2).

“*Eles ficam trocando de psicólogo. Eles tiram e nossos filhos ficam sem o acompanhamento*” (Mãe 3).

“*Sempre que eu venho aqui na saúde_mental sou bem atendida, mas também a falta de comunicação deles me atrapalha, porque quando chegamos aqui eles falam que o médico não vai atender, que nem hoje*” (Mãe 4).

### Trajetórias percorridas

**Figura 2:**
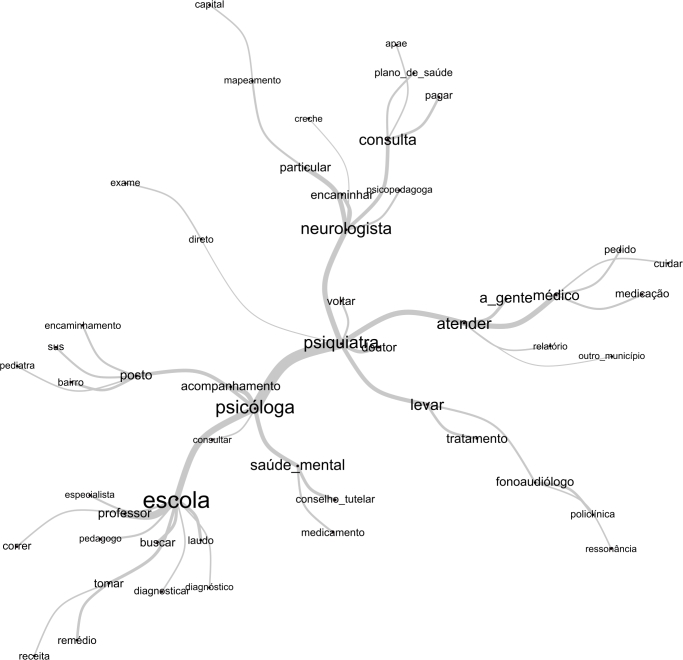
Grafo da análise de similitude gerado pelo software IRaMuTeQ, utilizando o *corpus* textual composto pela transcrição das entrevistas com os 14 genitores participantes, que ilustra os itinerários terapêuticos dos participantes.

Para finalizar, a Figura 2 exibe o grafo resultante da análise de similitude, o qual ilustra de modo específico a trajetória percorrida pelos participantes. Esse grafo expande as relações observadas na CHD, mostrando interações com outros profissionais e outros locais relevantes no processo de busca pelo cuidado em saúde mental infantojuvenil, como pediatras, Conselho Tutelar, Associação de Pais e Amigos dos Excepcionais (APAE), outros municípios e a capital do estado.

O grafo reforça o papel significativo da escola no reconhecimento inicial dos sinais e na busca por especialistas. “Neurologista” esteve mais relacionado a termos associados ao setor privado, além da conexão com o termo “capital”, indicando que alguns participantes buscaram exames especializados nessa região. O grafo também fortalece o sentido das UBS (“postinho do bairro”) no processo de obtenção de encaminhamentos para serviços especializados.

## Discussão

### Características dos usuários e cuidadores: reflexões a partir de dados coletados

Mesmo considerando a limitação metodológica de uma amostra por conveniência, observou-se que as prevalências de usuários do sexo masculino, em idade escolar correspondente ao Ensino Fundamental I, bem como de cor preta e parda, estão em consonância com estudos epidemiológicos realizados em serviços de saúde mental infantojuvenil ^15^ ^,^ ^16^. A prevalência de mulheres como principais cuidadoras e responsáveis nas interações com o serviço também é encontrada em outros estudos ^17^.

O predomínio de mulheres como principais cuidadores e acompanhantes nos serviços de saúde mental infantojuvenil reflete a persistência da feminização do cuidado no setor da saúde mental ^18^. Cuidar de crianças, especialmente daquelas que necessitam de serviços especializados em saúde mental, é uma tarefa intrinsecamente exigente. Considerando que a discussão sobre redes de apoio é crucial nessa área, é pertinente que os serviços estejam sensibilizados para reconhecer e responder aos desafios enfrentados por essas mulheres, oferecendo ações para apoiar seu bem-estar próprio, e não somente de seus filhos ^17^. A formação de grupos de apoio além de ações em sala de espera são exemplos de estratégias possíveis.

Sobre o aspecto diagnóstico, a prevalência de demandas que envolvem suspeita ou confirmação de transtornos do neurodesenvolvimento, como TEA e TDAH, também é uma tendência confirmada em estudos nacionais ^19^ ^,^ ^20^ e internacionais ^21^. No entanto, é importante problematizar essa crescente prevalência, considerando que a inclusão dos transtornos do neurodesenvolvimento na 5ª edição do Manual Diagnóstico e Estatístico de Transtornos Mentais (DSM-5) e a ênfase na biologia como base para esses diagnósticos podem estar contribuindo para uma cultura de hiperdiagnóstico e levando a um aumento das demandas e dos encaminhamentos de crianças e adolescentes. O uso generalizado desses diagnósticos, além de questionável do ponto de vista científico, pode aumentar o estigma, perpetuar práticas de medicalização desnecessárias e resultar em piores desfechos a longo prazo, como uma maior dependência de tratamentos farmacológicos e o reforço de uma identidade patológica desde a infância ^22^.

No âmbito da saúde mental infantojuvenil brasileira, persistem importantes questionamentos sobre como o sistema público de saúde deve se organizar para atender especificamente à demanda de crianças e adolescentes com TEA ^23^. Duas diretrizes ministeriais de 2013 apresentam abordagens distintas. A primeira relaciona o autismo ao campo das deficiências, enfatizando a reabilitação como forma de terapia ^24^. A segunda aborda o TEA como um transtorno mental, priorizando o cuidado dentro da RAPS, com especial foco nos CAPSij ^25^.

Um estudo recente investigou as barreiras ao acesso a 14 CAPSij do Rio de Janeiro ^26^. Identificou que os horários de funcionamento do serviço, a percepção de frequência insuficiente dos atendimentos, condições de acesso, logística e as más condições das instalações foram barreiras significativas ao acesso ao cuidado para TEA. Em contrapartida, outros estudos ressaltaram benefícios proporcionados pelos CAPSij, como melhorias na socialização e na criação de laços e aumento da autonomia - aspectos centrais na política pública. Entretanto, observam que familiares tendem a expressar preocupações sobre a abordagem generalista do serviço, solicitando um enfoque mais especializado no tratamento do autismo ^27^ ^,^ ^28^.

Em nosso estudo, as crianças e os adolescentes com TEA são atendidas no serviço Saúde Mental, assim como outros casos, principalmente por meio de consultas psiquiátricas e psicoterapia individual. Além disso, realizam acompanhamento fonoaudiológico e neurológico em serviços distintos, como a Policlínica e o CRE ou em rede privada. No entanto, observa-se predominância de abordagens individuais, centradas em avaliações e tratamentos especializados, sem explorar práticas mais amplas de cuidado, como o trabalho com grupos, oficinas terapêuticas e ações no território, as quais são previstas pela política da atenção psicossocial ^3^ ^,^ ^4^ ^,^ ^5^.

Por fim, destaca-se que o cenário de ambiguidade política, relacionado ao direcionamento de qual linha de cuidado adotar para crianças e adolescentes com TEA, também se apresenta no município deste estudo. No final de 2023, a prefeitura anunciou a criação do Centro Municipal de Atendimento ao Autista. Apesar de atender aos critérios estabelecidos para a implantação de um CAPSij, o município opta por implantar outro serviço que não está listado como ponto de atenção da RAPS para o cuidado em saúde mental infantojuvenil, o que merece uma reflexão.

Um espaço público focado para crianças e adolescentes com TEA tem sido intensamente reivindicado pelas Associações dos Amigos Autistas (AMA) do município. No entanto, a escolha de implementar esse serviço específico em detrimento de um CAPSij levanta preocupações quanto à sustentabilidade de uma solução isolada para responder a complexidade das necessidades de crianças e adolescentes com TEA, especialmente em uma região que parece ainda não possuir uma rede construída para o cuidado em saúde mental infantojuvenil de acordo com os princípios da atenção psicossocial. Além disso, emergem dúvidas sobre como os serviços, Saúde Mental e Centro Municipal de Atendimento ao Autista, serão organizados dentro da linha de cuidado e qual será o papel exato de cada um nessa rede. Questiona-se se ocorrerá um deslocamento do cuidado com demandas que envolvem TEA, sobreposição do cuidado ou se será estabelecida uma complementaridade coordenada e corresponsável.

### Potencialidades e desafios na identificação e encaminhamento

Estudos demonstram que a escola tem assumido um papel fundamental na identificação de possíveis necessidades em saúde mental, figurando como uma das principais fontes de encaminhamento para serviços ^29^. Esse fenômeno de encaminhamento de alunos para o setor de saúde mental é algo contemporâneo e se deve à ampliação das disciplinas “psi” e ao desenvolvimento de uma maior compreensão das relações entre possíveis problemas de aprendizagem e questões psicológicas, neurológicas e problemas no desenvolvimento. No entanto, esse processo está também intrinsecamente ligado a uma certa perspectiva sobre o sofrimento psíquico, caracterizada por uma abordagem medicalizante, calcada no diagnóstico e no tratamento de doenças e transtornos, o que frequentemente culmina na tríade patologia-tratamento-medicação.

O crescente aumento do discurso neurocientífico na educação pode levar a uma interpretação rígida de comportamentos desviantes de normas como sinais de transtornos, incentivando a busca precoce por diagnósticos e intervenções médicas, mesmo quando esses comportamentos poderiam ser parte de um desenvolvimento normal ou uma resposta contextual ^30^. Na prática, observa-se um cenário crescente de farmacologização da infância, fundamentado em um modelo centrado na doença, que postula que os medicamentos corrigem desequilíbrios neuroquímicos específicos. No entanto, esse modelo é amplamente criticado por sua base científica frágil e por desconsiderar a complexidade do desenvolvimento infantil e os significativos efeitos colaterais associados. Em vez de corrigir um suposto desequilíbrio neuroquímico, os medicamentos podem atuar mais como “intoxicantes cerebrais”, cujo principal efeito é a supressão dos sintomas por longos períodos de tempo ^31^.

Essa realidade ressalta a necessidade de um apoio mais amplo às equipes escolares, que possibilite uma compreensão mais contextualizada do fenômeno, incluindo a problematização das queixas e demandas endereçadas à saúde mental, a fim de evitar diagnósticos e encaminhamentos e pouco implicados, que acabam por reforçar abordagens medicalizantes e inadequadas. A escola, como principal espaço social no qual crianças e adolescentes passam a maior parte do tempo, torna-se o local onde muitos dos sinais de possíveis problemas de saúde mental se expressam. Esses sinais podem ser decorrentes de fatores diversos, e não só de condições neuropsicológicas, mas também de dinâmicas familiares conflituosas, vulnerabilidades socioeconômicas, racismo, exposição à violência, maus tratos, entre outros.

Embora a escola seja uma forte iniciadora da busca por cuidados em saúde mental infantojuvenil, essa função também tem gerado conflitos. Estudos criticam a escola por demandar à saúde a resolução de problemas de fracasso ou comportamento escolar sem repensar suas práticas e responsabilidades no fenômeno ^32^. No entanto, sabe-se que, em muitos casos, a escola não está preparada para lidar com a variedade e complexidade dos problemas de saúde mental, e pode entender que lidar com esses casos seria um desvio de suas funções pedagógicas.

Mesmo considerando a relevância dessas críticas, ainda é crucial reconhecer a escola como uma parceira essencial no cuidado. Em muitos casos, se não fosse a escola, importantes problemas biopsicossociais de crianças e adolescentes sequer seriam identificados, nem haveria impulso para a busca do suporte necessário ^29^.

O fortalecimento da relação entre escolas e serviços de saúde mental infantojuvenil requer um esforço conjunto. No entanto, os serviços devem ser proativos - uma vez que são eles que possuem um mandato público para cuidado em saúde -, buscando intensificar as ações intersetoriais e a colaboração com as instituições educacionais. É nesse sentido que se compreende a diretriz ministerial para o cuidado em saúde mental infantojuvenil: “*construção permanente da rede de intersetorialidade*” ^5^ (p. 25).

A colaboração entre os serviços de atenção e as escolas melhora a compreensão dos desafios enfrentados pelos educadores, fortalece o cuidado das infâncias e adolescências e promove corresponsabilização. Essa parceria evita a simplificação dos fenômenos de desenvolvimento infantil e processos de medicalização, além da culpabilização indevida de qualquer setor. Para tanto, é crucial valorizar espaços coletivos de discussão e análise, como fóruns temáticos, apoio matricial, reuniões intersetoriais e assembleias, os quais são frequentemente negligenciados pelas gestões municipais que priorizam o volume de atendimentos em detrimento do fortalecimento qualitativo da rede de cuidado ^29^.

Por outro lado, as UBS, comumente referenciadas pelos participantes como “postinho do bairro”, desempenharam um papel menos ativo no cuidado direto ao público infantojuvenil, e mais como intermediárias para serviços especializados. Nesse contexto, as UBS não emergiram primariamente como locais de cuidado inicial, mas como pontos de encaminhamento para especialistas.

Esse dado ilumina potencialmente três questões críticas. A primeira é o déficit de profissionais, em que muitas UBS sofrem com a escassez de profissionais diretamente ligados à especialidade da saúde mental e de modo acentuado à saúde mental infantojuvenil ^7^ ^,^ ^10^ ^,^ ^33^. Embora esse problema seja real e tenha impactos significativos, é essencial não simplificar a discussão apenas à “falta de profissionais especializados em saúde mental infantojuvenil” na atenção básica.

É verdadeiramente positivo que tais profissionais estejam presentes para um melhor cuidado em saúde mental infantojuvenil, mas é crucial ampliar o debate para um nível mais profundo. Nesse sentido, chega-se à segunda questão: a tendência das UBS em funcionar primordialmente como pontos de encaminhamento, em vez de locais de cuidado, pode também refletir uma falta de compreensão por parte das equipes sobre sua capacidade de conduzir atividades relacionadas ao cuidado em saúde mental infantojuvenil ^34^.

A ausência de psicólogos certamente limita a realização de avaliações psicológicas e psicoterapias; similarmente, a falta de psiquiatras restringe as avaliações psiquiátricas. No entanto, o acolhimento, a escuta ativa, a construção de uma demanda implicada, a realização de atividades de grupo, a orientação familiar e o trabalho em rede e com o território são exemplos de ações que podem ser realizadas por diferentes membros das equipes, como médicos, enfermeiros, técnicos de enfermagem e agentes comunitários de saúde ^35^. Além disso, embora a proposta da atenção básica não inclua psicólogos ou psiquiatras na equipe básica, esses profissionais estão presentes em serviços de atenção especializada, como os CAPS, e em equipes multiprofissionais, como o serviço Saúde Mental, que poderiam fortalecer o trabalho e o vínculo das famílias e crianças aos serviços de atenção básica, especialmente por meio do matriciamento ^36^.

Nesse sentido, chega-se à terceira questão crítica: a possível falha no estabelecimento de vínculo entre as famílias e as UBS. Como principal porta de entrada para a rede de cuidados e pela proximidade com o território, essas unidades são frequentemente acessadas para serviços como vacinação, cuidados médicos, odontológicos e de enfermagem ^35^. Idealmente, as equipes das UBS deveriam ser capazes de identificar necessidades de cuidado em saúde mental infantojuvenil, especialmente em situações de vulnerabilidade social e violência, além de serem vistas pelas famílias como pontos de apoio e cuidado, e não apenas como locais de encaminhamento ^36^.

### Reflexões sobre a trajetórias percorridas pelos familiares na busca pelo cuidado em saúde mental infantojuvenil no município

A descrição da busca por cuidado como um processo árduo, no qual as famílias precisam ser persistentes e ativas diante de um sistema que frequentemente impõe barreiras ao acesso, à continuidade e à regularidade do atendimento, não é exclusiva ao nosso estudo. Pesquisas de itinerários terapêuticos em saúde mental infantojuvenil anteriores descrevem a trajetória como um processo desgastante e de “peregrinação” ^12^. Durante o percurso, não é incomum que as famílias recorram a uma combinação de recursos dos sistemas de cuidado informais e formais. Os sistemas informais podem incluir práticas alternativas, com características místicas ou espirituais, enquanto os sistemas formais geralmente se referem aos serviços de saúde públicos e privados ^12^.

Contudo, em nosso estudo, não houve relatos sobre a utilização de práticas fora do sistema convencional de saúde. Por outro lado, houve utilização de serviços particulares, particularmente para especialidades que apresentaram longas filas de espera no Sistema Único de Saúde (SUS), como neurologia e fonoaudiologia, bem como a realização de exames mais relacionados à área da neurologia.

Embora tenham sido identificados desafios comuns relacionados à lentidão e à burocracia do sistema público, as experiências individuais variaram devido à qualidade do atendimento recebido. Muitos familiares reportaram interações positivas com profissionais dos serviços públicos, que demonstraram empatia e capacidade técnica. Trata-se de uma presença de suporte positivo dentro de um sistema desafiador. Isso porque, conforme indicado, a descontinuidade de atendimentos em razão de trocas de profissionais ou interrupções nos serviços prestados foram destacadas como barreiras significativas para o cuidado.

A alta rotatividade de profissionais da saúde é um dado recorrente em estudos em saúde mental infantojuvenil ^37^. Pesquisas identificam que profissionais da rede pública de saúde mental infantojuvenil relatam ter mais de um emprego para conseguir se sustentar e todos queixam-se da precarização e da falta de investimento nos serviços. Especialmente em municípios de pequeno e médio porte, incluindo os do interior, as contratações por meio de processos seletivos para contratos temporários são mais frequentes, e quando existem concursos para cargos efetivos, a remuneração é pouco atrativa ^38^.

A alta rotatividade de profissionais na saúde mental infantojuvenil gera uma série de consequências negativas para a qualidade do cuidado oferecido às infâncias e adolescências. Entre essas consequências, destacam-se a dificuldade na construção de vínculos terapêuticos e intersetoriais, a perda de *expertise* e do conhecimento territorial e o aumento da carga de trabalho para os profissionais remanescentes.

Um último aspecto importante a ser discutido é que nenhum dos participantes mencionou acompanhamento contínuo por serviços da atenção básica. Esse dado pode evidenciar uma possível desconexão ou subutilização desse nível da saúde no cuidado em saúde mental infantojuvenil.

Pesquisas indicam resistência das equipes da atenção básica em reconhecer e aproveitar seu potencial na saúde mental infantojuvenil, exacerbado pela falta de conhecimento específico, pouca afinidade pessoal ou profissional com o perfil infantojuvenil, sobrecarga de trabalho e suporte insuficiente da gestão e da rede especializada ^33^ ^,^ ^34^. Por outro lado, experiências positivas na atenção básica são reportadas, em que práticas focadas no fortalecimento do vínculo com o território, famílias e comunidade têm se mostrado potentes ^29^ ^,^ ^34^ ^,^ ^35^.

Destaca-se que o cuidado em saúde mental infantojuvenil na atenção básica não substitui a necessidade de atendimentos especializados quando necessários. No entanto, o encaminhamento deve ser visto não como um ato burocrático ou habitual, mas como um processo clínico implicado e corresponsável ^5^ ^,^ ^34^ ^,^ ^36^.

Apesar de focado em um território específico, este estudo reflete desafios comuns a muitos municípios brasileiros, independentemente do porte populacional ou da existência de redes implantadas. Nas cidades de pequeno e médio porte, especialmente aquelas localizadas no interior, há uma necessidade urgente de construir redes de cuidado desde o início ^10^ ^,^ ^11^. Em contraste, nas cidades maiores, o foco deve ser no fortalecimento das redes existentes. No entanto, a fragilidade do cuidado em saúde mental infantojuvenil é um problema que transcende essas diferenças regionais e alcança todos os municípios brasileiros.

Mesmo em cidades com redes mais robustas, a lógica do especialismo e da medicalização, bem como a fragilidade na operação intersetorial e na corresponsabilização, ainda imperam na organização do cuidado para essa população ^39^ ^,^ ^40^. Diante disso, é fundamental fortalecer a atenção psicossocial em todas as infâncias e adolescências, o que só pode ser alcançado por meio de uma política pública vigorosa, participativa e coletivamente avaliada. Superar a lógica da “consulta” e avançar para um cuidado que promova a corresponsabilização e a intersetorialidade é crucial para que a saúde mental infantojuvenil seja verdadeiramente presente na agenda política de todos os territórios.

## Considerações finais

Os itinerários terapêuticos de crianças e adolescentes tiveram as escolas como principais impulsionadoras na busca por cuidados e avaliações em outros serviços, enquanto as UBS atuaram principalmente como pontos de encaminhamento para especialistas. Espaços de atendimento particular foram frequentemente utilizados, especificamente para as especialidades de neurologia, psicologia e fonoaudiologia, enquanto o serviço Saúde Mental foi o serviço público predominantemente utilizado para o cuidado em saúde mental infantojuvenil no município. Recomenda-se que sejam feitos mais estudos incluindo a perspectiva dos profissionais e da gestão acerca dos processos de cuidado e sobre a disposição da RAPS do município para o cuidado em saúde mental infantojuvenil.
